# Magnetic Resonance Imaging for the Normal Mesostenium and Involvement of the Mesostenium in Acute Pancreatitis

**DOI:** 10.1155/2014/924845

**Published:** 2014-07-20

**Authors:** Xiao Xiao Chi, Xiao Ming Zhang, Tian Wu Chen, Wei Tang, Bo Xiao, Yi Fan Ji, Xiao Hua Huang

**Affiliations:** Sichuan Key Laboratory of Medical Imaging, Department of Radiology, Affiliated Hospital of North Sichuan Medical College, Nanchong, Sichuan Province 637000, China

## Abstract

The main point of this paper is to study MRI findings of the normal mesostenium and the involvement of the mesostenium in acute pancreatitis and to discuss the relationship between the involvement of the mesostenium and the severity of acute pancreatitis. In clinical practice, the mesenterical involvement in acute pancreatitis was often observed on MRI in daily works, which was little recorded in the reported studies. We conducted the current study to assess the mesenterical involvement in acute pancreatitis with MRI. We found that the mesenterical involvement of acute pancreatitis patients is common on MRI. The mesenterical involvement has a positive correlation with the MR severity index and the Acute Physiology and Chronic Healthy Evaluation II scoring system. It has been shown that MR can be used to visualize mesenterical involvement, which is a supplementary indicator in evaluating the severity of acute pancreatitis and local and systemic complications.

## 1. Introduction

The clinical morbidity of acute pancreatitis (AP) is high, and it has increased yearly [1]. AP is divided into mild and severe AP, with mortality of 15%–56% [2]. It originates from localized pancreatic and peripancreatic inflammation [3]. In cases in which the local protective responses fail, AP leads to extension of the local inflammatory mediators into the circulatory system, leading to systemic inflammatory response syndrome (SIRS) [4]. SIRS might cause multiple organ dysfunction and eventual multiorgan failure, which is associated with very high mortality [5]. Early diagnosis of AP and severe AP is essential to reducing mortality and improving the survival rate of AP.

The wide diffusion range of AP is a standard effect of the clinical severity of AP [6]. Early management of AP with local complications (pancreatic parenchymal necrosis and discrete fluid collection) is essential for the clinical assessment and establishment of the thresholds for specific interventions. In addition to the retroperitoneal space, acute pancreatitis could involve the subperitoneal space [7], which is very important in AP; however, there is less existing research on subperitoneal space involvement.

CT is the most rapid and effective imaging method for AP; however, the damage from CT radiation and the potential kidney toxicity from iodine have attracted increasing attention [8]. Iodine has been suspected of exacerbating pancreatitis [9], and the use of a CT enhanced scan should be determined based on the condition of the patient. CT is not sensitive to interstitial edema AP and mild AP [10]. MRI has an excellent resolution of soft tissue and could fully reflect pathological changes in AP [11]. With the continuing development of MR scanning techniques, such as diffusion-weighted imaging, MRI could facilitate the early diagnosis of AP [12]. MRI might be superior to CT for observing the mesenteries [13].

In this study, we performed MRI to observe normal findings of the mesenterical and its involvement in acute pancreatitis, and we discussed the relationship between the severity of mesostenium involvement and the severity of AP graded by the MR severity index (MRSI) and the Acute Physiology and Chronic Health Evaluation II (APACHE II) scores.

## 2. Materials and Methods

### 2.1. Patient Selection

This study was approved by the institutional review board of our hospital, and patient informed consent was waived. The observers were blinded to the clinical presentation, blood work, and outcomes of the patients.

The medical records and MRI images of the patients with acute pancreatitis admitted between January 2011 and November 2012 were reviewed retrospectively. The diagnosis of acute pancreatitis was based on the presence of typical abdominal pain combined with threefold elevated amylase or lipase. The inclusion criteria for the patients in this study were as follows: (a) acute onset of abdominal pain; (b) pancreatitis at the first onset; (c) threefold elevated amylase or lipase, excluding other causes of elevated enzymes; and (d) MR examination performed within 48 h after the onset of symptoms. The exclusion criteria in this study were as follows: (a) resistance to MR imaging; (b) a history of chronic pancreatitis; (c) AP caused by pancreatic carcinoma; and (d) mesenteric involvement induced by other diseases. A total of 196 patients with AP were recruited as our research subjects, including 96 men and 100 women with a mean age of 50 ± 12 years (range, 19–83 years). The patients had a laboratory workup and clinical assessment on admission.

Of 196 cases with AP, the etiology of AP was biliary in 105 patients, unknown in 37 patients, hyperlipidemia related in 33 patients, alcohol related in 12 patients, pregnancy related in 6 patients, and surgery related in 3 patients. A total of 127 patients had received a plain scan, whereas 69 patients had received a plain scan and a dynamic enhanced scan.

We selected the patients who had an upper-abdominal MR examination in our hospital from January to November 2011. A total of 806 cases were included. The inclusion criteria for the cases in this study were as follows: the patients had a plain scan and a dynamic enhanced examination. The exclusion criteria in this study were as follows: (a) resistance to MR imaging; (b) pancreatic disorders; (c) other diseases inducing mesenteric involvement such as hypoalbuminemia, malignant tumor, and abdominal cavity infection. A total of 50 patients were recruited as our research subjects. Twenty patients had no abnormal abdominal findings, 4 patients had a hepatic hemangioma, 15 patients had hepatic cysts, and 11 patients had renal cysts visualized on MRI. There were 24 males and 26 females, with a mean age of 51 ± 12 years (range, 28–80 years). The differences were not statistically significant for the age (*t* = 0.479, *P* = 0.633) and the sex ratio (*χ* ^2^ = 0.015,  *P* = 0.902) between the acute pancreatitis group and the control group.

### 2.2. MR Imaging Technique

The MR examinations were performed during suspended respiration with a 1.5-T system and a phased-array coil (Signa, GE Medical Systems, Milwaukee, WI, USA). The imaging sequences included an axial spoiled dual gradient-echo T1-weighted image (GRE T1WI), an axial respiratory-triggered fast recovery fast spin-echo T2-weighted image (FRFSE T2WI) with fat suppression, a coronal and axial single shot fast spin-echo T2-weighted image (SSFSE T2WI), SSFSE radial series slab MR cholangiopancreatography (MRCP), axial slab three-dimensional (3D) spoiled gradient-echo (SPGR) dynamic contrast-enhanced MR imaging with fat suppression, and an echo planar imaging diffusion weighted image (EPI-DWI). The parameters of the axial GRE T1WI were TR ms/TE ms = 150/2.7, flip angle = 90°, section thickness = 5~8 mm, and intersection gap = 0.5~1.0 mm. The parameters of FRFSE T2WI with fat suppression were TR ms/TE ms = 10,000~12,000/90~100 ms, section thickness = 5 mm, and intersection gap = 0.5 mm. The parameters of SSFSE T2WI were TR ms/TE ms = 2500~3500/80~100 ms, section thickness = 5 mm, and intersection gap = 0.5 mm. The parameters of MRCP were TR ms/TE ms = 6000/830~1100 ms and section thickness = 40~50 mm. The parameters of the axial three-dimensional SPGR dynamic MR image were TR ms/TE ms = 6.1/2.1, flip angle = 15~20°, and section thickness = 5 mm. The parameters of EPI-DWI were TR ms/TE ms = 1375/50, section thickness = 7 mm, and intersection gap = 1.5 mm. Gadolinium chelate was administered (0.2 mmol/kg) intravenously at approximately 3.5 mL/s by projector (Spectris MR Injection System, Medrad, Inc., USA) injection, followed by a 20 mL saline solution flush. Three enhanced phases were acquired. The first-pass arterial enhancement was optimized with a timing bolus sequence. The dynamic imaging was performed during breath holding before the injection (unenhanced), immediately after the injection (hepatic arterial phase), 30 s after the injection (early venous phase), 1 min after the injection (late venous phase), and 90 s after the injection (delayed phase).

### 2.3. MRI Images Interpretation

The original MRI data were loaded onto a workstation (GE, AW 4.4) for observation and measurement. The MRI images were reviewed by two observers (with more than 4 years of experience in abdominal MR imaging) who were blinded to the laboratory data and clinical outcomes.

#### 2.3.1. Normal Mesostenium on MRI

The normal mesenterical features observed on the MRI included the signal of the mesenterical adipose tissue (uniformity and consistency with the subcutaneous fat signal on the T2-weighted image); the shape of the superior mesenteric artery and vein (SMA and SMV); the shape and direction of the largest jejunal draining veins and the ileocolic artery; the total number of jejunal and ileal arteries on the dynamic enhanced MRI; the lymph nodes attached to the mesostenium and the root of the mesostenium (if larger than 5 mm in diameter).

#### 2.3.2. MRI Findings in Acute Pancreatitis on MRI

AP was graded as mild (0–3 points), moderate (4–6 points), or severe (7–10 points) according to the MR severity index (MRSI), which was derived from the CT severity index [14].

AP could involve the mesostenium including mesenterical edema, effusion, and involvement of the mesenteric vessels. The severity of mesenterical involvement (MI) was graded as Grade I (no abnormalities and mesenterical vessel involvement, 0 points); Grade II (a linear and patchy signal in the mesostenium, 1 point); or Grade III (mesenterical effusion, 2 points). We divided the mesostenium into right and left by the centerline of the body. In the cases in which the two sides of the MI score were not uniform, we selected the higher score as the final score.

### 2.4. The APACHE II Score

In clinical practice, the physician typically uses the APACHE II to evaluate the severity of acute pancreatitis [15]. AP was graded as mild (0–7 points) and severe AP (≥8 points), according to the APACHE II scoring system. An APACHE II score ≥8 points indicated much higher rates of morbidity and mortality [16]. The APACHE II score of the 196 patients was calculated according to the laboratory data and clinical outcomes by two physicians with 4 years of experience treating digestive diseases.

### 2.5. Statistical Analysis

The MR image data were averaged between the two observers. The interrater reliability was assessed using the kappa statistic.

The mean ± SD and range were used to express the continuous variables. Chi-squared tests were used to assess the differences in mesenterical involvement between mild and severe AP according to the APACHE II scoring system and between mild, moderate, and severe AP according to the MRSI. To correlate the MI with the MRSI and the APACHE II score, the Chi-squared tests and Spearman's rank correlation coefficient were used.

The statistical tests were performed using the Statistical Package for Social Sciences (SPSS) for Windows (Version 13.0, Chicago, IL, USA). *P* values <0.05 were considered significant.

## 3. Results

### 3.1. Normal Mesenterical Findings on MRI

The agreement between the observers for the mesenterical findings was good (*κ* = 0.816, *P* < 0.001).

The signal intensity of the mesenterical adipose tissue was uniform and consistent with the signal intensity of subcutaneous fat on the T2-weighted image.

**Figure 1:**
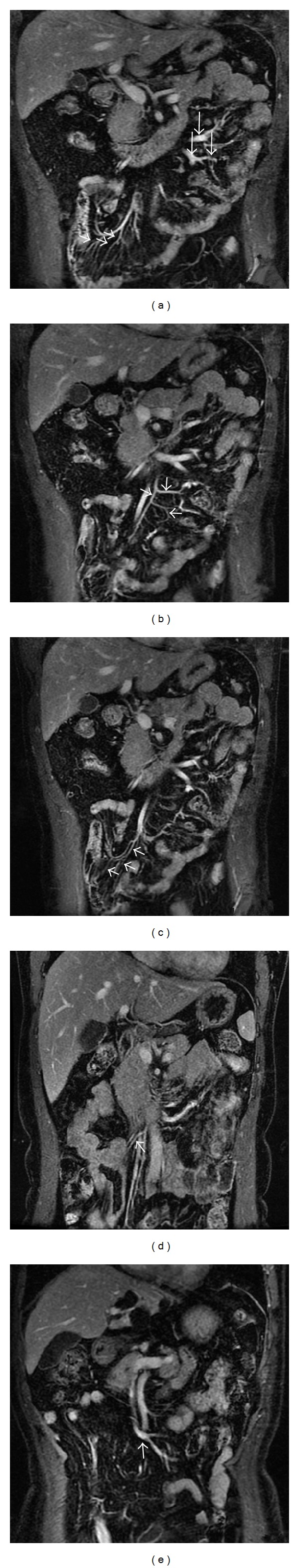
The jejunal veins (long arrow) and ileal veins (short arrow) showed enhancement on the coronal image (a), the jejunal arteries (b), the ileal arteries (c), and the ileocolic artery (d) showed enhancement on the coronal image. The largest jejunal draining veins showed enhancement on the coronal image (e).

There were 49 cases in which the SMA and SMV were in front of the aorta abdominalis, anteroinferior to the line feed, and left-right concomitant; one case was front-back concomitant. The display rates of the jejunal and ileal arteries were 100.0% in the axial and coronal view. In the coronal view, the larger branch vessels of the mesostenium course naturally and appear to be wavelike. The mesenterical vessels appear to be radially distributed and the blood vessel edges are clear; the diameter of the mesenterical blood vessels is smaller. In the axial view, the mesenterical vessels could be located in front of or behind the corresponding intestine and could appear to be round, oval, and oblong. The coronal view could spontaneously show the line feed of the trunk of the jejunal and ileal arteries, the aortic arch, and the straight arterioles, which could help to determine the location of the mesostenium. The trunk of the jejunal and ileal arteries appears to be to the left of the inferior line feed and to discharge into the left side of the SMA; the jejunal and ileal veins discharge into the SMV. In the 50 patients, the numbers of jejunal and ileal arteries (Figure 1) were not identical. There were 5 branches in 22% (11/50) of the patients, 6 branches in 20% (10/50) of the patients, 7 branches in 32% (16/50) of the patients, 8 branches in 20% (10/50) of the patients, and 9 branches in 6% (3/50) of the patients. The display rates were 100% in the coronal view of the ileocolic artery, which appeared to be to the right of the inferior line feed and to discharge into the right side of the SMA (Figure 1). The diameter of the ileocolic artery was 2.54 ± 0.35 mm (1.9 mm~3.39 mmm). The largest jejunal draining vein appeared to be a transverse trip vessel that discharged into the left side of the SMV (Figure 1). In the 50 patients, the diameter of the largest jejunal draining vein was 5 mm–10 mm (in 90% of the patients, 45/50), less than 5 mm (in 8% of the patients, 4/50), and greater than or equal to 10 mm (in 2% of the patients, 1/50). This vein could be observed in the early and late venous phases.

The mesenterical vessels demonstrated hypointensity on T2WI compared with fat and showed marked enhancement.

There were no lymph nodes larger than 5 mm in the mesostenium. However, 6% of the lymph nodes (3/50) were displayed at the root of the mesostenium, 10% (5/50) were displayed in the mesostenium, and 2% (1/50) were displayed at the identical time.

### 3.2. Findings of Acute Pancreatitis

The agreement between the observers for the MRSI was good (*κ* = 0.781,  *P* < 0.001). Among the 196 AP patients, the mean MRSI score was 4.14 ± 1.81 (ranging from 1 to 10). According to the MRSI, 37.2% (73/196), 54.1% (106/196), and 8.7% of the patients (17/196) had mild, moderate, and severe AP, respectively. Of the 196 AP patients, 140 patients (71.4%) were diagnosed with edematous AP, while 56 patients (28.6%) were diagnosed with necrotizing AP on MRI. Among the 56 patients with necrotizing AP, 39 had necrosis of less than 30% of the total pancreatic volume, 16 had necrosis of 30% to 50%, and 1 had necrosis of more than 50%. The mean APACHE II score was 5.20 ± 3.68 points (ranging from 0 to 21 points). A total of 152 patients were diagnosed with mild AP (77.6%), while 44 patients were diagnosed with severe acute pancreatitis (22.4%), according to the APACHE II scoring system.

### 3.3. Findings of Mesenterical Involvement on MRI

**Figure 2:**
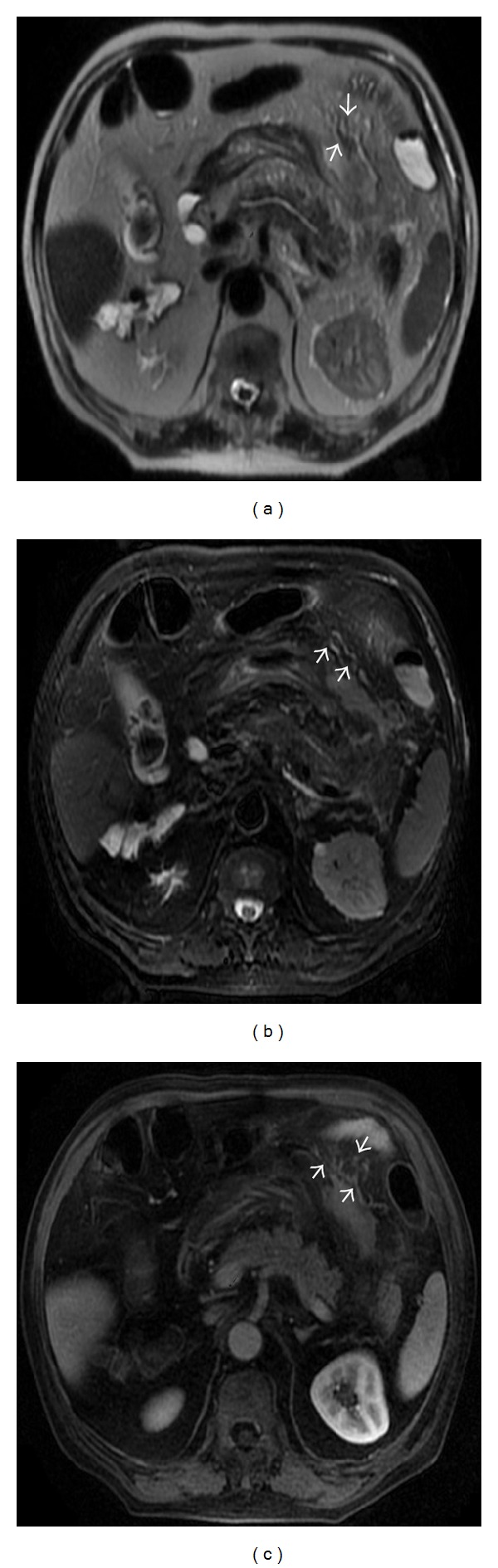
A 53-year-old male with mild AP. Mesenterical-vessel involvement could be seen on the axial SSFSE T2-weighted image (a) and on the axial SSFSE T2-weighted image with fat suppression (b). Mesenterical vessel showed the vessel-lumen expansion and dilated and tortuous lateral-branch small vessels on enhancement of the axial image (c).

**Figure 3:**
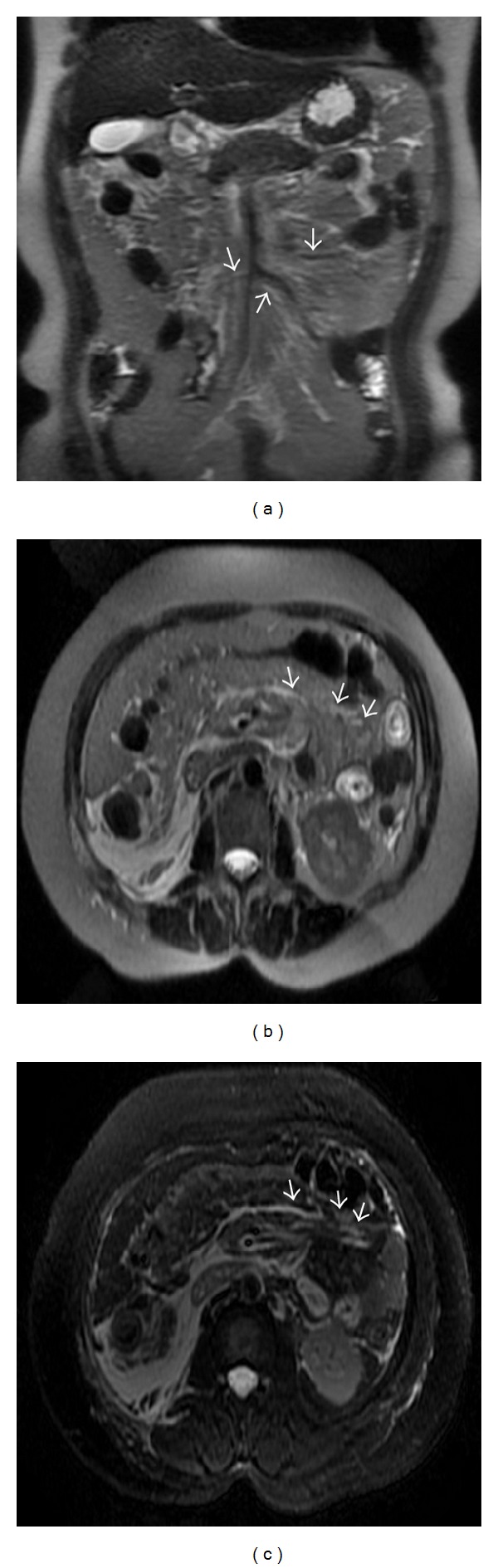
A 45-year-old male with moderate AP. The mesenterical edema and thickening on coronal SSFSE T2-weighted image (a), on axial SSFSE T2-weighted image (b), and on axial SSFSE T2-weighted with fat suppression image (c).

**Figure 4:**
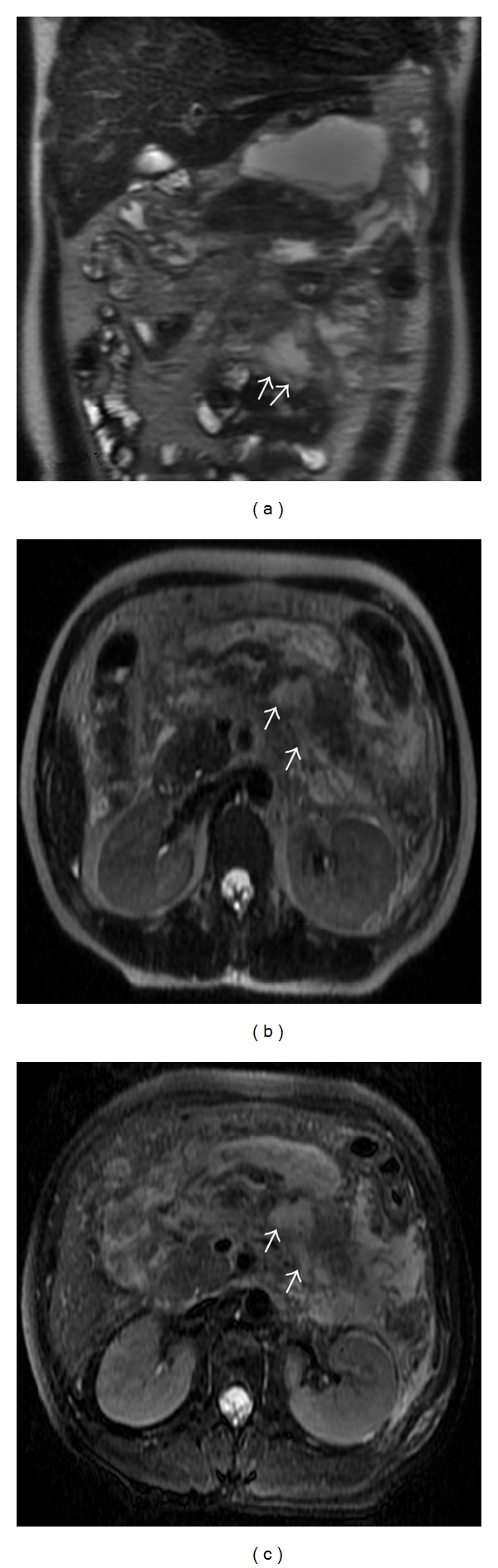
A 43-year-old female with severe AP. The mesostenium showed edema, thickening, and effusions on a coronal SSFSE T2-weighted image (a), on axial SSFSE T2-weighted image (b), and on axial SSFSE T2-weighted with fat suppression image (c).

AP involved the mesenterical vessels, which manifested blood-vessel-edge unsharpness, vessel-wall thickening, vessel-lumen expansion, and dilated and tortuous lateral-branch small vessels (Figure 2). There were 133 patients (67.9%) who had mesenterical-vessel involvement. The T1WI did not easily show the mesenterical edema and thickening, but it obviously was shown as linear or patchy high signal that was consistent with the running of the mesenteric vessels on T2WI and T2WI + FS (Figure 3). The effusions were shown as low signal on T1WI, and oval or patchy high signal on T2WI and T2WI + FS (Figure 4). According to the MI scoring system, there were 88 cases (44.9%), 58 cases (29.6%), and 50 cases (25.5%) in Grades I, II, and III, respectively.

### 3.4. The Relationship between Mesenterical Involvement and the MRSI

**Figure 5:**
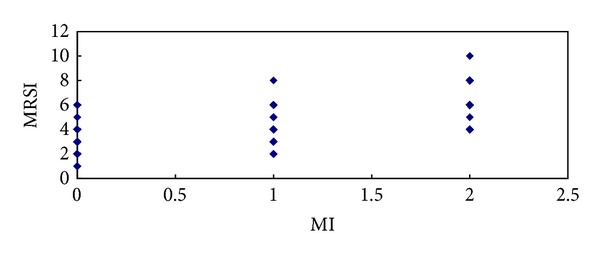
The correlation between the MRSI and MI.

Of the 196 patients with AP, 133 (67.9%) showed mesenterical involvement on MRI. In total, 133 patients had mesenterical-vessel involvement. The rates of mesenterical-vessel involvement in mild, moderate, and severe AP, respectively, were 31.5% (23/73), 87.7% (93/106), and 100% (17/17) (*χ* ^2^ = 71.481, *P* = 0.000). As shown in specific detail in Table 1, we found that the increase in the MRSI score was associated with the increase in the MI grade. Among the cases of mesenterical effusion, 8 cases occurred in the left mesostenium, 4 cases occurred in the right mesostenium, and 38 cases occurred on both sides. The MI on MRI was strongly correlated with the MRSI score (*r* = 0.722, *P* = 0.000) (Figure 5).

### 3.5. The Relationship between Mesenterical Involvement and the APACHE II Score

**Figure 6:**
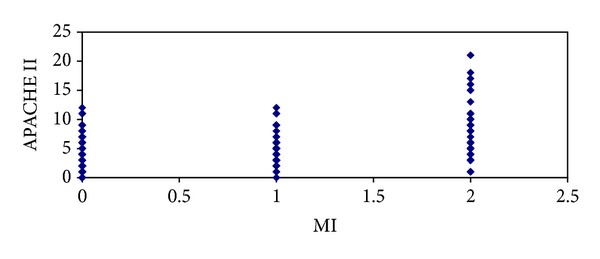
The correlation between the APACHE II and MI.

The percentage of mesenterical-vessel involvement in mild and severe AP was 64.5% (98/152) and 84.1% (37/44), respectively (*χ* ^2^ = 6.126,  *P* = 0.013). As shown in specific detail in Table 2, we found that the increase in the APACHE II score was associated with the increase in the MI grade. The mesenterical involvement observed on MRI was correlated with the APACHE II score (*r* = 0.364, *P* = 0.000) (Figure 6).

## 4. Discussion

In this study, we found that the mesenterical vessels could be visualized well on MRI; the display rates of the jejunal and ileal arteries were 100.0% in the axial and coronal views. The largest jejunal draining veins, 90% (45/50) of which had a diameter of 5 mm–10 mm, were a good indicator of the mesojejunum. The ileocolic artery was the terminal branch, and its display rate was 100% in the coronal view. The MI observed by MRI was strongly correlated with the MRSI score (*r* = 0.722) and the APACHE II score (*r* = 0.364). Our results indicated that MI could reflect local and systemic complications. Mesenterical effusion could serve as a supplementary indicator of the severity of AP. This finding might be important for the assessment of AP severity.

The mesostenium is a section of the subperitoneal space, and it includes blood vessels, lymphatic vessels, lymph nodes, nerve tissue, and adipose tissues [7]. The root of the mesostenium, which is connected to the anterior pararenal space and part of the mesentery and ligament of the abdominal cavity, contains the SMA and SMV, including their branches. The mesenterical root was associated with the start of the portal vein and the hepatoduodenal ligament on the right and was associated bilaterally with the ascending and descending colon by connective tissue; it was associated with the root of the transverse mesocolon in the front, and the iconic intersection vessel was the gastrocolic trunk [17]. The state of the mesentery artery and vein might signal the need to examine the mesentery [18]. Imaging of the SMA, SMV, the largest jejunal draining veins, and the ileocolic could help determine the position of the mesostenium and discriminate the mesostenium from the transverse mesocolon. In this study, the mesenterical vessel of the normal mesentery coursed naturally, and the edge was clear. The coronal view could display the entire range of the mesenterical vessels in several sections, and the image is spontaneous and the associative perception is strong.

Because the mesostenium is attached to the pancreatic body and tail junction, inflammatory substances and chemical enzymes diffuse along the mesostenium in cases of acute pancreatitis, which causes a slight increase in mesenteric vascular permeability and mesenterical edema. The MR images show a linear or patchy high signal, which is consistent with the course of the mesenteric vessels on the T2WI and T2WI + FS and the blood vessel-edge unsharpness. The adipose tissue and blood vessels in the mesentery showed in obvious contrast on the MRI, and the vessel lumen-expansion and tortuous lateral branch small vessels were clearly visible. In our study, mesenterical effusions occurred more on the left than on the right, possibly because AP typically occurs in the pancreatic body and tail. According to our results, 67.9% of the patients showed mesenterical involvement on MRI, which was higher than the percentage reported in the literature [19], indicating that 15.6% of the patients who had received a CT scan had mesentery involvement. It might be that CT was not sensitive to AP; however, MRI could accurately evaluate inflammatory lesions [20]. Our results indicated that MR is a good tool for visualizing the normal mesostenium and the involvement of the mesostenium in acute pancreatitis.

MRI is a reliable method for grading the severity of acute pancreatitis, and it has a prognostic value [21]. The MRSI is widely used to predict local complications and determine prognoses. Our results showed that classification of the incidence of MI increased with the addition of the MRSI score. With an increase in the APACHE II score, which reflects increased systemic complications, the general condition of the patient is observed as more serious [22]. In this study, MI was strongly correlated with the MRSI and APACHE II scores, and it could reflect local and systemic complications.

Our study had several limitations. First, the etiology of the mesenterical involvement was not acute pancreatitis. However, we had eliminated the possible causes of the mesenterical involvement by the exclusion criteria in our study. The prevalence of mesenterical involvement was high, and it was correlated with the MRSI. Second, the laboratory values of the APACHE II score were measured by several physicians and nurses, which might lead to variations between the observers. However, these variations might not affect the MR observation of the mesenterical involvement or the major results of this study.

MRI could be used to visualize the normal mesostenium and mesenterical involvement in acute pancreatitis. Mesenterical involvement plays an important role in evaluating the severity of AP and might reflect local and systemic complications. Mesenterical effusion could serve as a supplementary indicator of the severity of AP.

## Figures and Tables

**Table 1:** Mesenterical involvement and MRSI in AP.

MI Grade	Mild (*n* = 78)	Moderate (*n* = 114)	Severe (*n* = 18)	*P*	*χ* ^2^
Grade I	64	29	0	0.000	75.807
Grade II	14	50	2	0.000	18.199
Grade III	0	35	16	0.000	68.427

Chi-squared tests were used to analyze the differences between mild, moderate, and severe AP according to the MRSI in Grades I, II, and III MI.

**Table 2:** Mesenterical involvement and the APACHE II scoring system in patients with AP.

MI Grade	Mild (*n* = 163)	Severe (*n* = 47)	*P*	*χ* ^2^
Grade I	82	11	0.001	10.701
Grade II	54	12	0.323	0.977
Grade III	27	24	0.000	23.614

Chi-squared tests were used to analyze the differences between mild AP and severe AP according to the APACHE II scoring system in Grades I, II, and III MI.
